# Investigating the Relationship Between Entity Financialization, Managers’ Incentives, and Enterprise’s Innovation: Fresh Evidence From China

**DOI:** 10.3389/fpsyg.2021.810294

**Published:** 2022-03-04

**Authors:** Chaohui Xu, Haikuan Zhang, Mansi Wang, Amir Iqbal

**Affiliations:** ^1^School of Economics and Management, Hubei University of Science and Technology, Xianning, China; ^2^School of Management, Guangzhou University, Guangzhou, China; ^3^School of Innovation and Entrepreneurship, Entrepreneurship Institute, Guangzhou University, Guangzhou, China; ^4^School of Economics and Management, East China University of Technology, Nanchang, China

**Keywords:** financialization, enterprise’s innovation, manager’s compensation incentives, manager’s equity incentive, high-tech enterprise

## Abstract

The current study examines the relationship between financialization, managers’ incentives, and the enterprise’s innovation. Based on the principal-agent and incentive theories, this study proposes a research model with two management incentives as moderating variables between financialization and the enterprise’s innovation. First, we analyze the direct relationship between financialization and the enterprise’s innovation. Second, we examine the moderating effect of managers’ equity incentive and compensation incentives on the relationship between entity financialization and the enterprise’s innovation in high-tech/non-high-tech enterprises and state-owned and non-state-owned enterprises. This study covers the most recent updated data from both A-share listed companies in the Shenzhen and Shanghai stock exchange in China from 2009 to 2019. This study’s finding indicates a significant negative impact of entity financialization and the enterprise’s innovation. It means that the entity financial has a significant “crowding-out” effect on the enterprise’s innovation. This study also confirms that management incentives cannot effectively suppress a “crowding-out” impact of entity financialization on firm innovation because of the principal-agent severe problem in financialization. Finally, considering the heterogeneities of property rights and degrees of dependence on the enterprise’s innovation, a “crowding-out” effect of entity financialization on the enterprise’s innovation is more significant in high-tech and state-owned enterprises. Managers’ equity incentive significantly affects the enterprise’s innovation in high-tech enterprises, while the managers’ compensation incentive affects the enterprise’s innovation in state-owned enterprises. Our study could help the enterprise to improve the company manager’s incentive and provide the optimal assets allocation to improve the enterprise’s innovation ability. Lastly, this study provides significant policies and recommendations for the public sector high-tech enterprise and private sector high-tech enterprises. Moreover, policies and recommendations are fruitful for the public sector non-high-tech enterprise and private sector non-high-tech enterprise.

## Introduction

Since the big event, i.e., China Economic Reform and Open Up in 1978, China’s economy kept rapidly developing, attracting attention to other countries worldwide. In 2010, China became the world’s second-largest economy.^1^ There is no doubt that high-tech enterprises play an essential role in the Chinese economy (Liu et al., 2020). To furtherly promote economic transformation and development, the Chinese government has formulated a series of preferential measures to encourage enterprises to declare high-tech enterprises (Zhu Y. et al., 2021). Chinese high-tech enterprises refer to resident enterprises registered in China (excluding Hong Kong, Macao, and Taiwan) that continue to carry out research and development and transformation of technological innovations in the “high-tech industry” (Chen, 2011). In the last three decades, Chinese high-tech enterprises have played a critical role in bringing enterprises innovation (Cao et al., 2014). Similarly, Chinese non-high-tech enterprises are also a backbend of the Chinese economy. There is no doubt that high-tech and non-high-tech industries play a vital role in any country’s economic growth. However, both industries required massive investment for the enterprise’s innovation. Prior studies indicate that technological enterprises need extensive investments that positively enhance the enterprise’s innovation (Hsu et al., 2014; Kerr and Nanda, 2015; Pellegrino and Savona, 2017). Lall (1992) demonstrates that the enterprise’s innovation increases corporate financing and entrepreneurial assets. So, the financial assets also improve the innovation among the enterprises (Dosi, 1988; Jo, 2020). Thus, the relationship between enterprise investment and enterprise innovation becomes more important, and it attracts more attention from the managers and researchers.

Standing on the perspective of the enterprise’s financial structure, enterprise investment could be classified into physical investment and financial investment. With the increase of profits from financial investment, enterprises have a strong willingness to increase their investment in financial assets and to increase the proportion of financial assets in the enterprise’s capital structure. Therefore, the investment in production operation will be reduced. Hence, the phenomenon, i.e., entity financialization, is becoming more and more popular. A well-known conclusion is that financial assets inhibit the enterprise’s innovation when financialization was excessive in the 1990s (Dosi et al., 2016; Lee et al., 2020). Prior academic researchers also verify that unnecessary financialization will reduce the enterprise’s innovation (Mazzucato, 2013; Dosi et al., 2016), especially, enterprises’ basic research innovation will be reduced. Aristizabal-Ramirez et al. (2017) analyze the empirical data and find a negative correlation between financial development and the enterprise’s innovation. The main reason is due to the separation of ownership and management according to the perspective of enterprise governance.

The principal-agent theory is popularly applied in the enterprise’s hierarchical structure. Principal-agent risk is easy to appear because of the following three main reasons. Firstly, contracts are incomplete. Secondly, the shareholders’ responsibilities and the managers’ responsibilities are unequal. Thirdly, the tendencies of interests are not consistent. Thus, managers prefer to chase short-term investments, i.e., financial investment, for short-term gains rather than long-term gains. However, an enterprise’s innovation usually needs a long time to gain profits, financial investment, i.e., financialization, could cause managers’ shortsightedness, which causes managers to ignore the enterprise’s basic innovation (Dosi et al., 2016; Lee et al., 2020). Fortunately, managers’ incentive is a useful solution to improve the enterprise’s management. The appropriate manager’s incentive is beneficial for enhancing the corporate governance structure and optimizing the enterprise’s resource allocation (Wang H. et al., 2021). In a sense, the manager’s incentive could help the enterprise balance entity financialization and enterprise’s innovation. Managers’ incentive mainly includes the compensation incentive and equity incentive. Managers’ compensation incentive was popular several decades ago, and managers’ equity incentive is brought into the spotlight in recent years in China. According to the data from *Tonghuashun Data Center* (Chinese data center), the number of executives who hold their enterprises’ shares is increasing. The phenomenon that the employees hold shares is more popular. Therefore, equity incentive becomes an important role in managers’ decisions on rational allocation of enterprise resources.

This paper studies the relationship between entity financialization, managers’ incentive, and enterprise’s innovation based on previous analysis. We try to answer three questions.

RQ1: *Does entity financialization inhibit an enterprise’s innovation?*
RQ2: *Does managers’ equity incentive and compensation incentive influence the entity financialization and the enterprise’s innovation, respectively?*
RQ3: *What are the differences about relationships among entity financialization, manager’s incentive, and enterprise’s innovation in different property rights and innovation dependence?*

In addition, there are two innovations in this paper. Firstly, two moderating variables, i.e., manager’s equity incentives and compensation incentives, are introduced in this paper as we know until now. Compared with the previous studies, the moderating effect of manager’s equity and compensation incentives on the relationship between financialization and the enterprise’s innovation is first discussed. This innovation enriches the research field of entity financialization and helps enterprises optimize financial assets allocation and improve their innovative ability. Secondly, because of the obvious heterogeneity of the principal-agent conflicts of Chinese enterprises with the different property rights, we divide the enterprises into state-owned enterprises and non-state-owned enterprises according to the nature of enterprise property rights. The production technology of high-tech enterprises is complex and has a high dependence on innovation. Non-high-tech enterprises have a low technology demand and low innovation dependence. Thus, enterprises are divided into high-tech and non-high-tech enterprises groups according to their different dependencies of innovation. Subsequently, we, respectively, discuss two issues in each group, i.e., the relationship between the entity financialization and the enterprise’s innovation and a moderating effect of the manager’s equity incentive and compensation incentive on the connection above.

In summary, this paper has three theoretical contributions described as follows. Firstly, we enrich the research on financialization and provide a viewpoint to theoretically balance financial assets and entity product innovation investment for listed companies. Secondly, we provide suggestions for internal corporate governance by reducing the principal-agent risk. Thirdly, we expand the studies on the relationship between entity financialization and enterprise’s innovation. Moreover, this paper has three contributions in practice. Firstly, it could help enterprises optimize the allocation of their resources to achieve the maximum profit. Secondly, it could help enterprises balance investment in financial investment and the enterprise’s innovation, which could help the enterprises obtain a long-term profit and achieve sustainable development. Thirdly, it could help enterprises improve the ability of their internal corporate governance.

The rest of this paper is organized as follows. We review the previous literature in section “Literature Review”. Section “Methodology” provides the data, the model specification, and the estimation method. Section “Results and Discussion” provides the empirical results. Conclusion and policy recommendations are provided in Section “Conclusion, Policy Recommendation, and Future Works.”

## Literature Review

### The Concept of Financialization

Financialization is an arresting economic phenomenon and increasing influence of finance on the economy (Crotty, 2005; Krippner, 2005; Lapavitsas, 2011). Until now, the definitions of financialization have been different among researchers. However, it is no doubt that the proportion of corporate profits from the financial investment will stimulate financial investment activities in entity enterprises. The relationship between an enterprise’s physical investment and financialization has been studied by many researchers (Stockhammer, 2004; Crotty, 2005; Orhangazi, 2008; Seo et al., 2016). Furthermore, Krippner (2005) pointed that the enterprise with financialization mainly depends on financial channels to obtain profits rather than producing products for markets. Palley (2013) shows that financialization leads to an increase in financial investment, and resources will switch from physical investment to financial investment.

Thus, there are two different opinions about the relationship between financialization and enterprises’ development. Most scholars show that financialization has a negative impact on enterprises’ development. Over-financialization cannot fully achieve the optimal allocation of resources, and it is likely to decrease production yield and other problems. Dore (2008) shows that over-financialization would mismatch and waste resources. Ortiz (2014) argues that financialization is like a virus. It could achieve self-replication and reinforcement by grabbing enterprises’ internal resources, the development of the enterprise’s other departments will be inhibited. Palley (2013) discusses that financialization slows down the growth of the real economy and inflates the debt. Over-financialization makes enterprises switch from physical investment to financial investment. The excessive financial investment will take up enterprises’ limited resources, and then make capital of profit-making production inadequate and the enterprise’s innovation ability decrease. Consequently, the enterprise’s technology innovation will be difficult to be achieved. However, a lot of literature reveals that entity financialization is not always negative, but has positive effects on enterprises’ development in some situations. As the financial assets are able to deal with the risk of uncertainty of the external environment, hedge the volatility risk of the price and exchange rate, and alleviate enterprises’ financing difficulties, financialization also could promote the enterprise’s physical investment (Bloom et al., 2007). Furthermore, the investment in financial assets could cushion a sharp decline of profit of the enterprise’s main business (Baud and Chiapello, 2015). Some other scholars show that financial assets play the role of “reservoir,” in the enterprise’s daily operation, financial assets could reduce the cost when the enterprise is in financial plight, and encourage the enterprise’s innovation. Song and Lu (2015) analyze the data of China’s A-share listed enterprises from 2007 to 2012 and demonstrate that all enterprises tend to keep more financial assets. Zhong and Chen (2020) employ Youngor Group Co., Ltd. as a case study. They confirm that the enterprise’s decisions, such as financialization, strategic investment, and establishing a strategic partnership with the financial organization, could help the enterprise easily obtain credit loans, develop important business with loans, and provide financial support to improve the enterprise’s core competitiveness (Botzem and Dobusch, 2017). Cai et al. (2019) conclude that the leverage ratio of enterprises would be increased whatever the financialization is, and financial risks are usually reduced with the increase of the enterprise financialization.

### Financialization and Innovation

Since the financial crisis in 2008, the “real business” trend has stepped into the “virtual business,” and vast physical capital has switched to financial capital. The effect of financialization on the enterprise’s main business is getting more and more. In recent years, a growing number of scholars begin to focus on the relationship between entity financialization and the enterprise’s innovation. However, a consistent conclusion has not been achieved yet, there are two different viewpoints.

Firstly, some researchers agree that entity financialization promotes the enterprise’s innovation. Theurillat et al. (2010) find that increasing the proportion of financial assets in enterprises is beneficial for the efficiency of improving the enterprise’s asset allocation, which could reduce their financing difficulties and smooth the enterprise’s daily business operation. When financialization reaches the turning point value, enterprises will accelerate product innovation. The financial assets have strong liquidity, a high profit rate, and low transactional costs. When enterprises are short of research and development funds, enterprises could sell transactional financial assets to solve capital constraints and insufficient investment in the enterprise’s innovation. Thus, entity financialization could promote the enterprise’s innovation (Rasool et al., 2019). Liu and Zhang (2017) show that financial assets are conducive to promoting enterprises’ R&D and innovation in the future. Amin et al. (2020) find that transactional financial assets could alleviate financing constraints of private enterprises, reduce the R&D risk because of insufficient funds, and improve the stability and sustainability of the enterprise’s innovation. Li et al. (2019) find that entity financialization could improve the enterprise’s performance in the short term. When enterprises have insufficient capitals in operation, they could finance by selling their financial assets. It could reduce the risk of rupture in the enterprise’s capital chain, improve enterprises’ financing efficiency. Hence, financialization promotes the enterprise’s innovation. According to the empirical evidence, Demir (2009) demonstrates that diversified financial investments will bring enterprises high returns when the external environment is stable. When the economic environment is uncertain, risks are high. The fact that enterprises decide to invest in short-term financial assets could improve the profitability of enterprises and avoid enterprises falling into plight.

Secondly, based on previous studies, entity financialization not only promotes the enterprise’s innovation but also hinders the enterprise’s innovation. Some studies conclude that entity financialization prevents the enterprise’s innovation. As the resources that enterprises could obtain are limited, the enterprises do not only need to hire a large number of senior researchers for R&D, but also need a large amount of available liquidity. If the enterprise increases, the investment in financial markets, the enterprise’s investment in the main business, and the enterprise’s innovation will be reduced. Therefore, financialization has an inhibitory effect on the enterprise’s innovation. Lazonick (2010) found that financialization seriously “crowed-out” the investment in the enterprise’s main business and reduced the enterprise’s innovation. Orhangazi (2008) showed that financialization has a negative impact on the enterprise’s investment in the main business. With the increase of return of finance and speculation opportunities, enterprises constantly invest in the financial market and “crowd-out” the enterprise’s physical investment.

For instance, financialization “crowding-out” enterprise’s innovation is more significant in the Chinese state-owned enterprises. Zhang and Zhang (2016) found that financialization is significantly and negatively correlated with the investment rate of non-financial enterprises in physical assets. Bresnihan (2016) proved that financialization has an inhibitory effect on technological innovation ability with the empirical study. Zhu G. et al. (2021) found that holding the non-monetary financial asset negatively affects the enterprise’s innovation. Wang B. et al. (2021) argued that financialization significantly reduces the rate of industrial investment, since the monetary policy weakens the prosperity of the real economy and mismatches the financial assets, then the industrial investment will be “crowded out.” According to the empirical study of arbitrage motivation, Wang B. et al. (2021) proved that cross-industry arbitrage from the financial and real estate industry has a significantly negative correlation with the enterprise’s innovation. The results showed that cross-industry arbitrage significantly reduces the entity enterprise’s innovation. The stronger the corporate arbitrage motive, the greater the impact.

According to the previous research, the enterprise’s innovation is a long-term process, both the cost and risks of innovation are usually high. Because of the resource constraint, a “substitution” relationship exists between financial investment and physical investment. Thus, the investment in financial assets will occupy the capital for the enterprise’s innovation. In addition, according to the Optimal Sequence Financing Theory (OSFT), when an enterprise suffers financing constraints, the enterprise’s priority financing is internal financing. The enterprise uses its own financial assets to obtain profit and save transaction costs. Thus, financialization will “crowed out” the enterprise’s R&D investment, enterprise’s technological innovation would be decreased. With the increasing profits of financial assets, the enterprise’s capital structure will gradually change. When an enterprise could keep benefiting more from financial investment, an enterprise will continue investing in financial assets and reducing the enterprise’s innovation. Based on the above analysis, we propose the following hypothesis:

*Hypothesis 1: Entity financialization has a significant crowd-out effect on the enterprise’s innovation.*

### Manager’s Incentive and Financialization

Preferences of enterprise decision-makers determine the holding amount of financial assets. Financial assets have the characters of high-profit rates and strong liquidity. If managers focus on short-term performance, they will have a strong incentive to invest in financial assets. Many researchers have studied the relationship between managers’ characters and financialization. For example, Narayanan (1985) found that US managers prefer to obtain short-term benefits while giving up the long-term interests of shareholders. Campbell (2016) studied managers’ personal experience and financial decisions and found that managers who ever served in the army or underwent the great depression, made their financial decisions, which were significantly related to their previous experiences. Wright et al. (2013) showed that managers who experienced the financial crisis are more confident and have much stronger profit motives. They prefer to hold both the short- and long-term financial assets and use the different advantages of the two kinds of financial assets to improve the financialization. Some studies show that the equity incentive is a useful method to change the manager’s performance. With the implementation of equity incentives, enterprises’ managers become a dual status in the enterprise, which are both the operator and shareholder. Thus, their interests are closely consistent with the interests of enterprises. When they are making investment decisions, managers will pay more attention to their enterprises’ long-term profits. Dechow and Sloan (1991) showed that the increase in the proportion of senior executive shareholding would increase the enterprise’s R&D investment. In the final phase of CEO tenure, the CEOs who hold the equities do not prefer to reduce the enterprise’s R&D expenditure, but the CEOs who do not hold the equities will reduce their enterprises’ innovation expenditure. Yamada (2018) found that if equity incentive is applied to executives, enterprises’ R&D expenditure will significantly increase when managers have the power to decide enterprises’ R&D investment. Liu and Liu (2007) showed that the CEOs’ shareholding positively impacts enterprises’ R&D expenditure, and the implementation of CEOs’ equity incentives increases enterprises’ R&D investment. Some researchers continue to analyze the reasons that the effect of equity incentives on the enterprise’s R&D investment. Xu (2021) found that financialization has a “crowding-out” effect on R&D investment, and the implementation of equity incentives will decrease the negative effect of financialization on R&D investment. Basak et al. (2007) explained that equity incentive could inhibit managers to obtain profit from financial market and change the way of enterprises’ asset allocation. They also found that the effect of equity incentive on financial assets is not significant in technology-intensive industry, but significant in capital- and labor-intensive industries. Overall, equity incentive is a long-term incentive and a useful way for enterprise sustainable strategy. It could encourage managers to pay more attention to the enterprise’s performance and long-term development. Therefore, the mangers should reduce financialization and pay more attention to the “fundament” of enterprises’ development, i.e., enterprises’ innovation.

**FIGURE 1:**
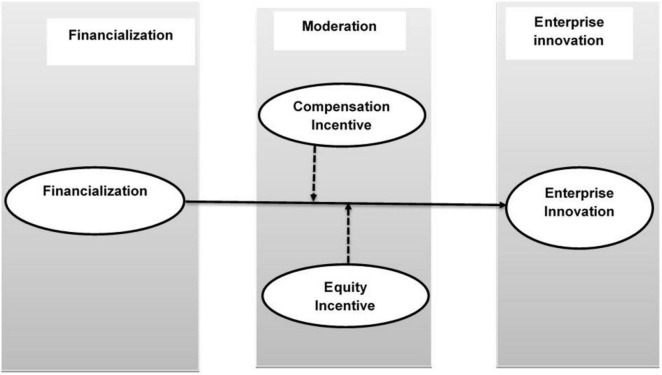
Research model. Dotted lines show a moderating relationship and solid lines indicate a direct relationship.

Compensation incentive is another useful and popular means to change a manager’s operation decision. A manager who implements compensation incentives will pay more attention to the enterprise’s short-term profits, which will result in the manager’s myopia, and the resources allocation for financial assets will be increased. Correspondingly, resources allocation for the enterprise’s main business will be reduced. The enterprise’s innovation will also be inhibited. Jensen and Meckling (1976) pointed out that the agency costs between managers and shareholders will cause managers to care only about their own interests rather than shareholders’ interests, such as agency’s misappropriating funds, stealing, and selling the enterprise’s core technology, and using the surplus funds for ineffective investment rather than paying cash dividends to shareholders. Yang et al. (2018) explained that compensation incentives would irritate mangers’ motivation for chasing profit from the financial market and significantly increase the enterprise’s financial asset allocation. Holmstrom and Weiss’s (1985) study has shown that because managers’ compensation is related to the performance of enterprises if managers’ efforts at work cannot be quantified, managers can quickly pay more attention to performance in a short time. As a result, managers are more willing to invest in financial assets. Agrawal and Mandelker (1992) explained that no matter whether managers decide to invest in the financial investment or in the enterprise’s innovation investment, they tend to choose the investment plans that maximize personal utility over those of shareholders, often against the interests of shareholders. Though many researchers focused on the effect of the manager’s incentive on the entity financialization or the enterprise’s innovation according to the previous studies. A few researchers studied the relationship among entity financialization, compensation incentive, and the enterprise’s innovation. Therefore, we took the manager’s incentives as the moderating variables between financialization and the enterprise’s innovation in this study. Based on the above analysis, we propose the following hypothesis. Similarly, the comprehensive research model of this study describes in Figure 1.

*Hypothesis 2: Managers’ equity incentive could reduce entity financialization’s “crowding-out” effect on enterprise’s innovation. But managers’ compensation incentives could increase.*

## Methodology

### Data

To examine the moderating effect of managers’ equity incentives, managers’ compensation incentives between financialization and the enterprise’s innovation, we used the panel data from Shenzhen and Shanghai A-share listed companies from 2009 to 2019. The data in this paper are mainly from the China Stock Market and Accounting Research Database (CSMAR), which is collected and operated by the company, Guotaian. Guotaian is the first and largest professional high-tech company that engages in the design and development of accurate financial and economic information databases in China. Considering the impact of the financial crisis in 2008 on the capital market, to keep the data consistent, the data were truncated from listed companies in the capital market after the financial crisis. In the data processing, we followed the three ways. Firstly, we deleted the data of the *S.T.* and *S.T. listed companies as the financial data of such companies were marked as abnormal by China Securities Regulatory Commission (CSRC).^2^ Secondly, the financial listed companies were excluded. Thirdly, the data of the sample of the incomplete and extreme values were excluded. Consequently, 8,189 samples from 2,261 listed companies were collected.

### Variables

In this paper, we selected 10 variables to examine the impact of financialization on enterprises’ innovation. These variables are separated into independent, moderators, control, and dependent variables. The study used enterprises’ innovation as the dependent variable, managers’ equity incentives and manager compensation incentives as the moderating variables, and financialization as independent variables. This study also chooses the board of directors, large shareholders, a growth rate of total assets, company size, capital expenditures, financial leverage, and profitability as controlled variables. Table 1 shows every variable, its symbol, and its measurement units.

**TABLE 1:** Description of variables.

Variables	Symbol	Categories	Proxy used and measurement units	Source
The enterprise’s innovation	*Invention*	Dependent variable	Measured by the logarithmic of the number of invention patents plus 1	Database of CSMAR in China
Financialization	*Fin*	Independent variable	Measured by the proportion of financial asset allocation, and the proportion of financial asset allocation = (trading financial assets + derivative financial assets + net loans and advances + net saleable financial assets + net holdings to maturity investments + Financial assets in other current assets and long-term equity investments + net investment real estate)/total assets	
Compensation incentive	*Salary*	moderator variables	Calculated as the natural logarithm of the managers’ total monetary compensation in a year	
Equity incentive	*Equity*		Calculated as the percentage of shares held by managers at the end of the year	
Board of directors	*Board*	Control variables	Expressed by the ratio of independent directors being divided by the number of board members	
Large shareholders	*Large*		Measured by the shareholding ratio of the largest shareholder	
Growth rate of total assets	*Growth*		Expressed in ratio that is the growth value of the company’s total assets is divided by the total assets at the end of the period	
Size of the company	*Size*		Expressed by the natural logarithm of the company’s total assets at the end of the period	
Capital expenditure	*Fix*		Expressed by the fixed ratio that the net asset is divided by the total assets at the end of the period	
Financial leverage	*Lev*		The ratio of liability being divided by total assets at the end of the period	
Profitability	*ROA*		Expressed in ratio that is annual net profit of the company is divided by total assets at the end of the period	

#### Dependent Variable

The enterprise’s innovation ability (*Invention*) is the dependent variable. The logarithmic of the number of invention patents is used to measure the innovation ability of listed companies.

#### Independent Variable

Financialization (*Fin*) is the independent variable. According to the classification of financial assets in previous literature, the proportion of financial asset allocation equals the sum of (trading financial assets + derivative financial assets + net loans and advances + net saleable financial assets + net holdings to maturity investments + financial assets in other current assets and long-term equity investments + net investment real estate) is divided by total assets.

#### Moderating Variables

Managers’ incentive (*Incentive*) is the moderating variable, including two kinds. The first kind is the managers’ compensation incentive (*Salary*), calculated as the natural logarithm of the managers’ total monetary compensation in a year. The second kind is managers’ equity incentive (*Equity*), which is calculated as the percentage of shares held by managers at the end of the year.

#### Control Variables

To elaborate, we introduce seven control variables into the abovementioned model, which are according to the financial characteristics and corporate governance. They are the board governance (*Board*), large shareholder governance (*Large*), the growth of the company (*Growth*), the size of the company (*Size*), capital expenditure (*Fix*) financial leverage (*Lev*), and profitability (*ROA*). In addition, the industry (*Industry*) and the year (*Year*) are also taken as control variables. Board governance (*Board*) is expressed by the ratio of independent directors being divided by the number of board members. Large shareholder governance (*Large*) is measured by the shareholding ratio of the largest shareholder. The company’s growth (Growth) is expressed in a ratio that is the growth value of the company’s total assets divided by the total assets at the end of the period. The company’s size (*Size*) is expressed by the natural logarithm of the company’s total assets at the end of the period. Capital expenditure (*Fix*) is expressed by the fixed ratio that the net assets are divided by the total assets at the end of the period. Financial leverage (*Lev*) indicates the ratio of liability being divided by total assets at the end of the period. Profitability (*ROA*) is expressed in a ratio that is an annual net profit of the company is divided by total assets at the end of the period. *Industry* and *years* are both represented by the dummy variables 0 and 1.

### Models

The moderating effect model is used in this paper. The moderating effect model has been widely accepted and popular applied by researchers in management. Entity financialization has a direct impact on the enterprise’s innovation. Managers, who are the decision-makers in the enterprises, could allocate enterprise resources and have greater power whether the enterprise invests in a financial asset or the enterprise’s innovation. The manager’s incentive has a moderating effect on the relationship between financialization and the enterprise’s innovation. Therefore, a moderating effect model is used in our studies. According to the abovementioned hypotheses and variables, the model is constructed as follows:


(1) $$\begin{array}{c} Invention_{i,t}=\alpha_{0}+\alpha_{1}Fin_{i,t}+\alpha_{2}Board_{i,t}+\alpha_{3}Large_{i,t}\\ \;+\alpha_{4}Growth_{i,t}+\alpha_{5}Size_{i,t}+\alpha_{6}Fix_{i,t}+\alpha_{7}Lev_{i,t}\\ \;+\alpha_{8}ROA+\sum Industry+\sum Year+\varepsilon \end{array}$$

(2) $$\begin{array}{c} Invention_{i,t}=\beta_{0}+\beta_{1}Fin_{i,t}+\beta_{2}Incentive_{i,t}+\beta_{3}Fin_{i,t}\\ \;\times Incentive_{i,t}+\beta_{4}Board_{i,t}+\beta_{5}Large_{i,t}\\ \;+\beta_{6}Growth_{i,t}+\beta_{7}Size_{i,t}+\beta_{8}Fix_{i,t}+\beta_{9}Lev_{i,t}\\ \;+\beta_{10}ROA+\sum Industry+\sum Year+\varepsilon . \end{array}$$

## Results and Discussion

### Descriptive Statistical Analysis

Table 2 shows the descriptive statistical results of the main variables. The average value of *Invention* is 1.2814, while its maximum and minimum values are 7.9306 and 0, respectively. The SD is 1.2578, indicating significant differences in enterprises’ innovation capacity among different listed companies in China. The average value of *Fin* is 0.0729, while its maximum and minimum values are 0.8121 and 0, respectively. The SD is 0.1052, which indicates many differences in financialization among different listed companies in China. Some listed companies even do not yet invest in financial investment. The average value of *Equity* is 0.1876, while its maximum and minimum values are 0.9822 and 0, respectively. It shows that the level of the manager’s equity incentives in Chinese listed companies is high and they are different among the different listed companies. The average value of *Salary* is 15.2457, while its maximum value is 18.7124 and the minimum value is 10.7144. It indicates minor differences in the manager’ compensation incentives among different listed companies in China. The mean value of the *board* is 0.3754, with an SD value of 0.0561. It indicates that the proportion of independent directors in the listed companies’ board is slightly more significant than the standard rules. Thus, the board governance needs to be strengthened. The mean value of *growth* is 0.2567 with an SD value of 0.5311, which indicates that the listed companies have a rapid growth rate. The maximum value is 8.8176, and the lowest value is −0.7253, indicating a difference in the *growth* among different listed companies. Some listed companies need to finance more for their business development. The mean value of *size* is 21.9250, with an SD value of 1.2911. It signifies that there is a slight difference among different companies. The maximum size is 28.6365 and the minimum value is 18.7215, which indicates that the difference among scales of the enterprises is small.

**TABLE 2:** Descriptive statistics.

Variable	Mean	Maximum	Minimum	SD
*Invention*	1.2814	7.9306	0.000	1.2578
*Fin*	0.0729	0.8121	0.000	0.1052
*Equity*	0.1876	0.9822	0.000	0.2240
*Salary*	15.2457	18.7124	10.7144	0.6888
*Lev*	0.3828	0.9925	0.0075	0.2010
*Size*	21.9250	28.6365	18.7215	1.2911
*Fix*	0.2090	0.9542	0.000	0.1402
*ROA*	0.0519	0.5872	–0.7436	0.0663
*Growth*	0.2567	8.8176	–0.7253	0.5311
*Large*	0.3474	0.8824	0.0220	0.1464
*Board*	0.3754	0.8000	0.1818	0.0561

*Source: Author’s constructed.*

According to the capital expenditure index (*Fix*), the mean and SD are 0.2090 and 0.1828, respectively, indicating a slight difference in capital expenditure among different listed companies. The maximum financial leverage (*Lev*) is 0.9925 and the minimum value is 0.0075. It indicates that the asset-liability ratio of sample enterprises is quite different. Some enterprises have a big debt ratio. The average value of *Lev* is 0.3828, which suggests that the debt ratios of sample enterprises are all high. About enterprises’ profitability, the maximum *ROA* is 0.5872, and the minimum value is −0.7436. The values indicate that some sample enterprises have a big net profit and strong profitability, while others have severe losses. The average value of the profitability is 0.0519, which suggests that the sample enterprises have a small growth rate of net profit. The ratios of the holding share of the largest shareholder (*Large*) are used to measure the major shareholder’s governance. The mean value of *Large* is 0.3474 with an SD value of 0.1464. It indicates that ratios of the holding share of the largest shareholders are significant; the phenomenon of “single-largest shareholder” is widespread in Chinese enterprises.

Table 3 shows the *Pearson-correlation* analysis results of the main variables. According to the results, *Pearson-correlation* coefficients of the main variables are all less than 0.8, which indicates that collinearity is less likely to exist in the linear regression.

**TABLE 3:** Pearson-correlation analysis.

Variable	*Invention*	*Fin*	*Equity*	*Salary*	*Board*	*FCF*	*Growth*	*Size*	*Fixed*	*Lev*	*ROA*
*Invention*	1										
*Fin*	−0.014	1									
*Equity*	−0.050***	0.011	1								
*Salary*	0.369***	−0.013	−0.090***	1							
*Board*	0.011	0.023	0.189***	−0.029	1						
*FCF*	0.063***	0.051***	−0.043**	0.175***	−0.026	1					
*Growth*	0.031*	−0.059***	0.046***	0.076***	0.009	−0.053***	1				
*Size*	0.434***	−0.014	−0.293***	0.507***	−0.030*	0.094***	0.027	1			
*Fixed*	−0.051***	−0.229***	−0.171***	−0.043**	−0.032*	0.164***	−0.174***	0.194***	1		
*Lev*	0.140***	−0.185***	−0.282***	0.101***	−0.043**	−0.133***	0.026	0.508***	0.223***	1	
*ROA*	0.060***	0.069***	0.088***	0.231***	−0.026	0.453***	0.262***	−0.037**	−0.228***	−0.363***	1

***Significance at the 1% level, **Significance at the 5% level, *Significance at the 10% level.

### Benchmark Regression Analysis

Table 4 shows the results of the effect of entity financialization on the enterprise’s innovation. The impact of *Fin* on *Invention* (the test path is “*Fin*?*Invention*”) is − 0.4012, and the value of *p* is 0.004. It indicates that financialization has a significant negative impact on the enterprise’s innovation at a 1% significance level. The value of − 0.4012 explains that with 1 unit increase in financialization, the enterprise’s innovation will decrease by 0.4012 units. In other words, financialization “crowed out” the enterprise’s innovation investment. When an enterprise’s financial investment is increased, the enterprise’s investment in innovation will be “crowded out.” Therefore, Hypothesis 1 is verified.

**TABLE 4:** Regression results of financialization and the enterprise’s innovation.

Items	Regression coefficients	S. E.	*T*-value	*P*-value
*Fin_*i, t*_*	−0.4012***	0.1391	−2.88	0.004
*Lev_*i, t*_*	−0.1220	0.0905	−1.35	0.178
*Size_*i, t*_*	0.2913***	0.0137	21.21	0.000
*Fix_*i, t*_*	−1.0195***	0.1096	−9.31	0.000
*ROA_*i, t*_*	0.3220	0.2246	1.43	0.152
*Growth_*i, t*_*	−0.0769***	0.0270	−2.85	0.004
*Large_*i, t*_*	−0.1193	0.0956	−1.25	0.212
*Board_*i, t*_*	0.7025***	0.2388	2.94	0.003
*Constant_*i, t*_*	−5.7262***	0.7468	−7.67	0.000
*Year/Industry*	Control			
*R* ^2^	0.1040			
*F*-value	31.67			
N	8,189			

***Significance at the 1% level, **Significance at the 5% level, *Significance at the 10% level.

Furthermore, the regression coefficient of company size on the enterprise’s innovation is 0.2913, and the value of *p* is 0.000, which indicates that the sample companies’ size significantly and positively affects the enterprise’s innovation at the 1% level of significance. The value of 0.2913 explains that with a 1 unit increase in company size, the enterprise’s innovation will increase by 0.2912 units. The larger the company is, the more the attention is paid to the company’s basic R&D. The regression coefficient of capital expenditure (*Fix*) on the enterprise’s innovation is −1.0195, and the value of *p* is 0.000; it shows that capital asset investment has squeezed out the enterprise’s innovation investment. The value of −1.0195 explains that with a 1 unit increase in capital expenditure, the enterprise’s innovation will decrease by 1.0195 units. The regression coefficient of enterprise growth on the enterprise’s innovation is −0.0769, and the *t*-value is −2.85. They show that sample enterprises’ growth negatively affects enterprises’ innovation. The value of −0.0769 explains that with a 1 unit increase in growth, the enterprise’s innovation will decrease by 0.0769 units. The regression coefficient of board governance on the enterprise’s innovation is 0.7025 and the value of *p* is 0.004, which indicates that board governance positively affects the enterprise’s innovation. The value of 0.7025 explains that with a 1 unit increase in the board of governance, the enterprise’s innovation will be increased by 0.7025 units. The better the corporate governance is, the greater the investment in the enterprise’s innovation will be. The improvement of board governance promotes the enterprise’s innovation. Therefore, Hypothesis 1 is verified.

### Robustness Test

#### Robust Test and Clustering Test

Table 5 shows the regression results of robust SE estimation and cluster SE estimation. They show that the regression coefficients of both *Fin* and *Invention* are −0.4012, which are significant at the 1 and 5% levels, respectively. Thus, the financialization of entity enterprises inhibits the enterprise’s innovation ability, which is consistent with the main test conclusion.

**TABLE 5:** Robust test and clustering test.

Items	Robust test			Cluster test		
	Regression coefficients	S. E.	*P*-value	Regression coefficients	S. E.	*P*-value
*Fin_*i, t*_*	−0.4012***	0.1348	0.003	−0.4012**	0.1812	0.027
*Lev_*i, t*_*	−0.1220	0.0921	0.185	−0.1220	0.1495	0.415
*Size_*i, t*_*	0.2913***	0.0183	0.000	0.2913***	0.0324	0.000
*Fix_*i, t*_*	−1.0195***	0.1104	0.000	−1.0195***	0.1740	0.000
*ROA_*i, t*_*	0.3220	0.2229	0.149	0.3220	0.2915	0.270
*Growth_*i, t*_*	−0.0769***	0.0252	0.002	−0.0769***	0.0280	0.006
*Large_*i, t*_*	−0.1193	0.0998	0.232	−0.1193	0.1610	0.459
*Board_*i, t*_*	0.7025***	0.2671	0.009	0.7025*	0.4005	0.080
*Constant_*i, t*_*	−5.7262***	0.7152	0.000	−5.7262***	0.8604	0.000
*Year/Industry*	Control			Control		
*R* ^2^	0.1074			0.1074		
*F*-value	22.19			12.22		
N	8,189			8,189		

***Significance at the 1% level, **Significance at the 5% level, *Significance at the 10% level.

#### Selected Alternative Key Variables

The total number of patents and the amount of R&D investment are used to replace the number of inventive patents, which express the enterprise’s innovation ability. The natural logarithm of the total number of patents is used to measure the enterprise’s innovation ability, expressed by *Patent*. The ratio, i.e., the enterprise’s innovation capability is expressed by R&D investment, which is measured by the amount of the R&D investment divided by the enterprise’s income. In Table 6, the regression results of the enterprise’s innovation indicators replaced are reported. When the dependent variable is the total number of patents and the independent variable is entity financialization, the regression coefficient is −0.3412. The value of *p* is 0.007, which is significant at the 1% level. When the dependent variable is R&D investment and the independent variable is entity financialization, the regression coefficient of entity financialization is −0.0094, and the value of *p* is 0.001, which is significant at the 1% level. There is no significant difference between the results and the previous regression analysis.

**TABLE 6:** Select alternative key variables.

Items	*Patent_*i, t*_*			*R and D_*i, t*_*		
	Regression coefficients	S. E.	*P*-value	Regression coefficients	S. E.	*P*-value
*Fin_*i, t*_*	−0.3412***	0.1262	0.007	−0.0094***	0.0028	0.001
*Lev_*i, t*_*	0.1372*	0.0821	0.095	−0.0530***	0.0019	0.000
*Size_*i, t*_*	0.5341***	0.0125	0.000	−0.0016***	0.0003	0.000
*Fix_*i, t*_*	−1.1954***	0.0994	0.000	−0.0441***	0.0023	0.000
*ROA_*i, t*_*	0.6381***	0.2037	0.002	−0.0554***	0.0044	0.000
*Growth_*i, t*_*	−0.0940***	0.0245	0.000	−0.0001	0.0006	0.939
*Large_*i, t*_*	0.3021***	0.0868	0.001	−0.0218***	0.0021	0.000
*Board_*i, t*_*	1.0901***	0.2167	0.000	0.0289***	0.0052	0.000
*Constant_*i, t*_*	−10.3621***	0.6776	0.000	0.0841***	0.0160	0.000
*Year/Industry*	Control			Control		
*R* ^2^	0.3041			0.2633		
*F* *-* *value*	116.44			228.28		
*N*	8,189			20,983		

***Significance at the 1% level, **Significance at the 5% level, *Significance at the 10% level.

#### Test of Endogenous Problems

Table 7 shows the test results of endogenous problems. The enterprise’s innovation may impact its financial asset, and it may result in endogenous problems caused by reverse causality. To solve the endogenous problem, our study will use the lag one period of *Fin* as the instrumental variable to test the regression results between the lag one period of *Fin* and the *Invention*, *Patient*, *R&D*. In Table 6, the consistency of instrumental variables is significantly negative, and there is a significant negative correlation between instrumental variables and endogenous variables. This is consistent with the abovementioned results, which show that there is still a “crowding-out” effect of enterprise financialization on the enterprise’s innovation when the endogenous problem is considered.

**TABLE 7:** Test of endogenous problems.

Items	*Invention_*i, t*_*			*Patent_*i, t*_*			*R and D_*i, t*_*		
	Regression coefficients	S.E.	*P*-value	Regression coefficients	S. E.	*P*-value	Regression coefficients	S. E.	*P*-value
*Fin*_i, t–1_	−0.6722***	0.1968	0.001	−0.3132*	0.1720	0.069	−0.0134***	0.0034	0.000
*Lev*_i, t–1_	−0.0519	0.1220	0.67	0.2312**	0.1066	0.030	−0.0509***	0.0022	0.000
*Size*_i, t–1_	0.3274***	0.0188	0.000	0.5380***	0.0164	0.000	−0.0010***	0.0003	0.003
*Fix*_i, t–1_	−1.2499***	0.1490	0.000	−1.2014***	0.1302	0.000	−0.0470***	0.0027	0.000
*ROA*_i, t–1_	0.2177	0.3249	0.503	0.4614	0.2839	0.104	−0.0423***	0.0056	0.000
*Growth*_i, t–1_	−0.0198	0.0368	0.589	−0.0230	0.0321	0.473	0.0022***	0.0007	0.001
*Large*_i, t–1_	−0.0902	0.1260	0.474	0.3566***	0.1101	0.001	−0.0211***	0.0024	0.000
*Board*_i, t–1_	1.0018***	0.3180	0.002	1.1497***	0.2779	0.000	0.0249***	0.0062	0.000
*Constant*_i, t–1_	−6.2463***	0.7981	0.000	−9.1620***	0.6974	0.000	0.0674***	0.0154	0.000
*Year/Industry*	Control			Control			Control		
*R* ^2^	0.1065			0.3051			0.3437		
*F*-value	18.95			67.13			303.99		
N	4,822			4,822			19,091		

***Significance at the 1% level, **Significance at the 5% level, *Significance at the 10% level.

### Further Analysis of the Moderating Effect of Manager Incentive Mechanism

Table 8 shows the manager’s incentive on the regression results of the enterprise’s innovation. The independent variable is the manager’s compensation incentive, i.e., *Salary*, and the dependent variable is the enterprise’s innovation, i.e., *Innovation*. Salary’s regression coefficient is 0.2005, and the value of *p* is 0.000, significant at the 1% level. They indicate that the manager’s compensation incentive could increase the enterprise’s innovation. However, the regression coefficient of *Equity* is −0.0527, and the value of *p* is 0.435. They suggest that it is not significant for the relationship between managers’ equity incentives and the enterprise’s innovation. The reason is that monetary compensation has a dominant effect, but equity incentive is only a tiny proportion compared to the compensation incentive of listed companies in China. Compared with equity incentive, compensation incentive has a more significant effect on the enterprise’s innovation.

**TABLE 8:** Managers’ incentive and corporate innovation.

Items	*Salary_*i, t*_*→*Invention*_i, t_			*Equity_*i, t*_*→*Invention*_i, t_		
	Regression coefficients	S. E.	*P*-value	Regression coefficients	S. E.	*P*-value
*Salary_*i, t*_*	0.2005***	0.0245	0.000			
*Equity_*i, t*_*				−0.0527	0.0675	0.435
*Lev_*i, t*_*	−0.0395	0.0883	0.655	−0.0752	0.0891	0.398
*Size_*i, t*_*	0.2270***	0.0155	0.000	0.2845***	0.0142	0.000
*Fix_*i, t*_*	−0.8777***	0.1080	0.000	−0.9713***	0.1083	0.000
*ROA_*i, t*_*	−0.0751	0.2290	0.743	0.3394	0.2254	0.132
*Growth_*i, t*_*	−0.0620**	0.0269	0.021	−0.0711***	0.0271	0.009
*Large_*i, t*_*	−0.0217	0.0959	0.821	−0.1181	0.0958	0.218
*Board_*i, t*_*	0.8165***	0.2384	0.001	0.7109***	0.2399	0.003
*Constant_*i, t*_*	−7.4691***	0.7744	0.000	−5.6415***	0.7527	0.000
*Year/Industry*	Control			Control		
*R* ^2^	0.1104			0.1032		
*F*-value	33.79			31.39		
N	8,189			8,189		

***Significance at the 1% level, **Significance at the 5% level, *Significance at the 10% level.

Table 9 shows the regression results of managers’ incentives on financialization. The independent variable is the manager’s compensation incentive, i.e., *Salary*, and the dependent variable is entity financialization, i.e., *Fin*, the regression coefficient of *Salary* is 0.0073, the value of *p* is 0.000, which is significant at the 1% level. They indicate that the manager’s compensation incentive increases the proportion of financial asset allocation. When the independent variable is the manager’s equity incentive, i.e., *Equity*, the regression coefficient of *Equity* is −0.0320, the value of *p* is 0.000, which is significant at the 1% level. They indicate that the manager’s equity incentive inhibits the entity’s financialization. It is found that the manager’s compensation incentive intensifies the entity’s financialization, while the manager’s equity incentive significantly inhibits the entity’s financialization. The reason is that the performance compensation system is popularly implemented in China’s listed companies, which indicates that the manager’s compensation is highly and positively related to their company’s performance. The financial assets are usually highly profitable in the short term; managers tend to increase the allocation of financial assets and improve the enterprise’s profitability in the short term, increasing their monetary compensations. Thus, the level of financialization is improved. However, the manager’s equity incentive is positively related to the enterprise value in the long term. Financialization is not conducive to increasing the enterprise’s competitiveness; it negatively affects its value in the long term. It only improves an enterprise’s performance in the short term, and it crowed out its productive investment and the enterprise’s innovation. Therefore, an equity incentive is conducive to long-term development as managers’ equity incentives could inhibit financialization and increase the enterprise’s innovation.

**TABLE 9:** Managers’ incentive and corporate financialization.

Items	*Salary_*i, t*_*→*Fin_*i, t*_*			*Equity_*i, t*_*→*Fin_*i, t*_*		
	Regression coefficients	S. E.	*P*-value	Regression coefficients	S. E.	*P*-value
*Salary_*i, t*_*	0.0073***	0.0017	0.000			
*Equity_*i, t*_*				−0.0320***	0.0052	0.000
*Lev_*i, t*_*	−0.1097***	0.0063	0.000	−0.1140***	0.0063	0.000
*Fix_*i, t*_*	−0.1314***	0.0086	0.000	−0.1388***	0.0086	0.000
*ROA_*i, t*_*	−0.0079	0.0184	0.665	0.0164	0.0178	0.358
*Growth_*i, t*_*	−0.0090***	0.0022	0.000	−0.0081***	0.0022	0.000
*Large_*i, t*_*	−0.0022	0.0075	0.773	−0.0078	0.0076	0.306
*Board_*i, t*_*	0.0299	0.0191	0.118	0.0360*	0.0191	0.060
*Constant_*i, t*_*	0.0456	0.0613	0.457	0.1615***	0.0559	0.004
*Year/Industry*	Control			Control		
*R* ^2^	0.1832			0.1852		
*F*-value	62.22			63.04		
N	8,189			8,189		

***Significance at the 1% level, **Significance at the 5% level, *Significance at the 10% level.

Table 10 shows the moderating effect of managers’ incentives on the relationship between entity financialization and the enterprise’s innovation ability. About the moderating effect of managers’ compensation incentive, the regression coefficient of the cross item (Salary * Fin) between manager’s compensation incentive and entity financialization is −0.1614, the value of *p* is 0.382. Hence, the moderating effect of manager’s compensation incentive on the relationship between entity financialization and the enterprise’s innovation ability is not significant, managers’ compensation incentive cannot effectively restrain a crowding-out effect of entity financialization on the enterprise’s innovation.

**TABLE 10:** The effect of manager’s incentive on financialization.

Items	Managers’ compensation incentive			Managers’ equity incentive		
	Regression coefficients	S. E.	*P*-value	Regression coefficients	S. E.	*P*-value
*Fin_*i, t*_*	2.0659	2.8264	0.465	−0.2769	0.1845	0.133
*Salary_*i, t*_*	0.2124***	0.0280	0.000			
Salary_*i, t*_ * Fin_*i, t*_	−0.1614	0.1846	0.382			
*Equity_*i, t*_*				−0.0191	0.0781	0.806
Equity_*i, t*_ * Fin_*i, t*_				−0.5669	0.5283	0.283
*Lev_*i, t*_*	−0.0947	0.0902	0.294	−0.1301	0.0911	0.154
*Size_*i, t*_*	0.2310***	0.0155	0.000	0.2880***	0.0142	0.000
*Fix_*i, t*_*	−0.9329***	0.1096	0.000	−1.0212***	0.1103	0.000
*ROA_*i, t*_*	−0.0810	0.2289	0.724	0.3391	0.2253	0.132
*Growth_*i, t*_*	−0.0647**	0.0269	0.016	−0.0744***	0.0271	0.006
*Large_*i, t*_*	−0.0280	0.0959	0.770	−0.1220	0.0958	0.203
*Board_*i, t*_*	0.8253***	0.2383	0.001	0.7160***	0.2399	0.003
*Constant_*i, t*_*	−7.6613***	0.8007	0.000	−5.6513***	0.7524	0.000
*Year/Industry*	Control			Control		
*R* ^2^	0.1112			0.1040		
*F*-value	32.05			29.81		
N	8,189			8,189		

***Significance at the 1% level, **Significance at the 5% level, *Significance at the 10% level.

According to the moderating effect of the manager’s equity incentive, the regression coefficient of the cross item (Equity*Fin) between manager’s equity incentive and entity financialization is −0.5669, the value of *p* is −0.283. Hence, the moderating effect of manager’s equity incentive on the relationship between entity financialization and the ability of the enterprise’s innovation is not significant, managers’ equity incentive cannot effectively restrain a “crowding-out” effect of entity financialization on the enterprise’s innovation. So, Hypothesis 2 could not be verified.

With the development of corporate governance by maximizing shareholder values and the application of manager’s incentives, managers prefer financial asset investment in order to improve the enterprise’s short-term performance (Sen and DasGupta, 2014). Financial assets have both liquidity and profitability, which provides convenience for managers’ selfish behavior. There will be a serious agency problem when the entity enterprise transfers to “virtual enterprise.”

### Heterogeneity Analysis

#### Differentiation of the Innovation Dependence of Enterprises

According to the different degrees of dependence of the enterprise’s innovation, entity enterprises are divided into high-tech enterprises and non-high-tech enterprises. The high-tech enterprises have complex production technology and a great demand for technology, and they have a strong dependence on innovation. However, the non-high-tech enterprises have complex production technology and a small demand for technology, and they have a weak dependence on innovation. In high-tech enterprises, technological innovation is more important than other production factors. Technological innovation is an important factor of core competitiveness in high-tech enterprises. According to document, i.e., the industry classification instruction of listed enterprises (2012), which is issued by CSRC, five industries are defined as high-tech enterprises, which includes pharmaceutical manufacturing, aviation spacecraft and equipment manufacturing, electronic and communication equipment manufacturing, computer and office equipment manufacturing, and medical equipment and instrument manufacturing. Otherwise, the other industries are defined as non-high-tech enterprises.

Tables 11, 12 show the regression results by distinguishing the dependence of innovation, manager’s incentive, and entity financialization on the enterprise’s innovation. In the high-tech enterprises, the regression coefficient of *Fin* is −0.4555 and the value of *p* is 0.029. In non-high-tech enterprises, the regression coefficient of *Fin* is −0.1985 and the value of *p* is 0.285. Compared with non-high-tech enterprises, the crowding-out effect of entity financialization on the enterprise’s innovation is only significant in technology-intensive enterprises. In non-high-tech enterprises, the importance of innovation is weak. Thus, the innovative ability is low in non-high-tech enterprises, and the allocation of financial assets has a weak impact on the enterprise’s innovation. In technology-intensive enterprises, innovation is important. The allocation of financial assets will squeeze the enterprise’s innovation. According to the cross items in Tables 11, 12, only the manager’s equity incentive in high-tech enterprises lessens the crowding-out effect of entity financialization on the enterprise’s innovation, it indicates that the manager’s incentive mechanism in listed companies is needed to be changed.

**TABLE 11:** Distinguishing dependence of innovation (high-tech enterprise).

Items	Regression coefficients	S. E.	*P*-value	Regression coefficients	S. E.	*P*-value	Regression coefficients	S. E.	*P*-value
*Fin_*i, t*_*	−0.4555**	0.2091	0.029	1.2958	4.2439	0.760	−0.1098	0.2816	0.697
*Salary_*i, t*_*				0.1971***	0.0473	0.000			
*Salary* *_*i, t*_* * Fin *_*i, t*_*				−0.1150	0.2775	0.679			
*Equity_*i, t*_*							0.0444	0.1166	0.703
*Equity* _*i, t*_ * Fin_*i, t*_							−1.4636*	0.7821	0.061
*Lev_*i, t*_*	−0.2114	0.1434	0.141	−0.1866	0.1432	0.193	−0.2189	0.1438	0.128
*Size_*i, t*_*	0.4312***	0.0235	0.000	0.3640***	0.0277	0.000	0.4274***	0.0245	0.000
*Fix_*i, t*_*	−0.7699***	0.2027	0.000	−0.7758***	0.2022	0.000	−0.7632***	0.2037	0.000
*ROA_*i, t*_*	−0.1799	0.3483	0.606	−0.5360	0.3560	0.132	−0.1555	0.3491	0.656
*Growth_*i, t*_*	−0.0993**	0.0400	0.013	−0.0863**	0.0400	0.031	−0.0993**	0.0402	0.014
*Large_*i, t*_*	−0.2064	0.1538	0.180	−0.1523	0.1538	0.322	−0.2058	0.1539	0.181
*Board_*i, t*_*	0.5604	0.3826	0.143	0.7219*	0.3833	0.060	0.5243	0.3848	0.173
*Constant_*i, t*_*	−8.1620***	0.5068	0.000	−9.7107***	0.6807	0.000	−8.0841***	0.5309	0.000
*Year/Industry*	Control			Control			Control		
*R* ^2^	0.1442			0.1489			0.1447		
*F*-value	33.32			31.18			30.20		
N	3,453			3,453			3,453		

***Significance at the 1% level, **Significance at the 5% level, *Significance at the 10% level.

**TABLE 12:** Distinguishing the dependence of innovation (non-high-tech enterprises).

Items	Regression coefficients	S. E.	*P*-value	Regression coefficients	S. E.	*P*-value	Regression coefficients	S. E.	*P*-value
*Fin_*i, t*_*	−0.1985	0.1857	0.285	2.6556	3.7679	0.481	−0.2386	0.2415	0.323
*Salary_*i, t*_*				0.1628***	0.0345	0.000			
Salary_*i, t*_ * Fin_*i, t*_				−0.1872	0.2459	0.447			
*Equity_*i, t*_*							−0.0036	0.1046	0.973
*Equity* _*i, t*_ * Fin_*i, t*_							0.1900	0.7157	0.791
*Lev_*i, t*_*	−0.0914	0.1151	0.427	−0.0658	0.1149	0.567	−0.0903	0.1164	0.438
*Size_*i, t*_*	0.2102***	0.0167	0.000	0.1691***	0.0187	0.000	0.2107***	0.0172	0.000
*Fix_*i, t*_*	−0.5574***	0.1371	0.000	−0.4950***	0.1373	0.000	−0.5587***	0.1382	0.000
*ROA_*i, t*_*	0.6734**	0.2900	0.020	0.3664	0.2958	0.216	0.6713**	0.2915	0.021
*Growth_*i, t*_*	−0.0508	0.0364	0.162	−0.0434	0.0364	0.233	−0.0517	0.0366	0.157
*Large_*i, t*_*	0.0530	0.1204	0.660	0.1303	0.1212	0.283	0.0531	0.1209	0.661
*Board_*i, t*_*	0.6871**	0.3009	0.022	0.7585**	0.3005	0.012	0.6821**	0.3026	0.024
*Constant_*i, t*_*	−3.8081***	0.7403	0.000	−5.3095***	0.8217	0.000	−3.8132***	0.7505	0.000
*Year/Industry*	Control			Control			Control		
*R* ^2^	0.0772			0.0817			0.0768		
*F*-value	13.78			13.77			12.94		
N	4,736			4,736			4,736		

***Significance at the 1% level, **Significance at the 5% level, *Significance at the 10% level.

#### Different Enterprise Property Rights

According to the obvious heterogeneity of principal-agent conflict and enterprise control right problem because of different property rights in state-owned and private enterprises, we further discuss the heterogeneous effect of financialization on the enterprise’s innovation in different property rights. Tables 13, 14 show the regression results of property heterogeneity. It shows that the coefficients of state-owned enterprises and private enterprises are significantly negative at the significance level of 10%, but the absolute value and *t*-value of the regression coefficient of state-owned enterprises are greater, which indicates that the state-owned enterprises may have a stronger crowding-out effect on the enterprise’s innovation investment. Because the senior managers in state-owned enterprises generally implement the permanent system, manager’s salary and promotion are closely related to the performance appraisal during their tenure. Due to the intertemporal and uncertain nature of innovation investment, a large amount of capital investment of senior executives during their tenure will only be a “wedding dress” for following or future executives. Therefore, the current management may not have enough enthusiasm to engage in innovation activities, but it may increase short-sighted behavior because of the pressure of short-term performance, thus managers will reduce or even give up the investment in long-term activities, such as R&D investment and innovation, and increase financial investment for short-time high returns. According to the analysis of cross items, considering the moderator effect of managers’ incentives, only the managers’ compensation incentive in state-owned enterprises inhibits the crowding-out effect of entity financialization on the enterprise’s innovation. Thus, the manager’s incentive mechanism does not effectively achieve the effect of entity financialization on the enterprise’s innovation.

**TABLE 13:** The regression results of property heterogeneity (state-owned enterprises).

Items	Regression coefficients	S. E.	*P*-value	Regression coefficients	S. E.	*P*-value	Regression coefficients	S. E.	*P*-value
*Fin_*i, t*_*	−0.6650*	0.3481	0.056	11.3230*	6.6692	0.090	−0.5169	0.3661	0.158
*Salary_*i, t*_*				0.2379***	0.0566	0.000			
Salary_*i, t*_ * Fin_*i, t*_				−0.7808*	0.4348	0.073			
*Equity_*i, t*_*							2.5309**	1.2065	0.036
*Equity_*i, t*_* * Fin_*i, t*_							−12.9403	13.1421	0.325
*Lev_*i, t*_*	−0.3086	0.1886	0.102	−0.2710	0.1882	0.150	−0.2533	0.1903	0.183
*Size_*i, t*_*	0.3200***	0.0260	0.000	0.2705***	0.0291	0.000	0.3197***	0.0260	0.000
*Fix_*i, t*_*	−1.4382***	0.1973	0.000	−1.3048***	0.1995	0.000	−1.4027***	0.1980	0.000
*ROA_*i, t*_*	0.6060	0.6156	0.325	−0.0962	0.6392	0.880	0.5832	0.6184	0.346
*Growth_*i, t*_*	−0.0968	0.0835	0.246	−0.1010	0.0833	0.225	−0.1275	0.0847	0.132
*Large_*i, t*_*	−0.4096	0.2017	0.042	−0.2981	0.2041	0.144	−0.3181	0.2084	0.127
*Board_*i, t*_*	1.1242**	0.4827	0.020	1.1726**	0.4817	0.015	1.1491**	0.4826	0.017
*Constant_*i, t*_*	−7.0005***	0.1594	0.000	−9.5343***	0.9717	0.000	−7.0851***	0.1594	0.000
*Year/Industry*	Control			Control			Control		
*R* ^2^	0.1630			0.1415			0.1369		
*F*-value	14.44			14.16			13.67		
N	2,477			2,477			2,477		

***Significance at the 1% level, **Significance at the 5% level, *Significance at the 10% level.

**TABLE 14:** Property heterogeneity (non-state-owned enterprises).

Items	Regression coefficients	S. E.	*P*-value	Regression coefficients	S. E.	*P*-value	Regression coefficients	S. E.	*P*-value
*Fin_*i, t*_*	−0.2679*	0.1460	0.067	−1.4954	3.0209	0.621	−0.0534	0.2145	0.803
*Salary_*i, t*_*				0.2052***	0.0322	0.000			
Salary_*i, t*_ * Fin_*i, t*_				0.0797	0.1974	0.687			
*Equity_*i, t*_*							0.0045	0.0808	0.956
*Equity_*i, t*_* * Fin_*i, t*_							−0.7696	0.5517	0.163
*Lev_*i, t*_*	−0.0245	0.1042	0.814	0.0083	0.1038	0.936	−0.0329	0.1044	0.753
*Size_*i, t*_*	0.2592***	0.0183	0.000	0.1819***	0.0209	0.000	0.2555***	0.0188	0.000
*Fix_*i, t*_*	−0.6376***	0.1340	0.000	−0.5976***	0.1334	0.000	−0.6365***	0.1349	0.000
*ROA_*i, t*_*	0.2801	0.2294	0.222	−0.0558	0.2327	0.811	0.2877	0.2300	0.211
*Growth_*i, t*_*	−0.0541**	0.0272	0.047	−0.0426	0.0271	0.116	−0.0525*	0.0272	0.054
*Large_*i, t*_*	0.0024	0.1096	0.983	0.0441	0.1092	0.686	0.0031	0.1097	0.977
*Board_*i, t*_*	0.2932	0.2747	0.286	0.3982	0.2738	0.146	0.2947	0.2755	0.285
*Constant_*i, t*_*	−4.4889***	0.1133	0.696	−6.0218***	0.1127	0.000	−4.4252***	0.7607	0.000
*Year/Industry*	Control			Control			Control		
*R* ^2^	0.0796			0.0883			0.0797		
*F*-value	16.92			17.77			15.98		
N	5,712			5,712			5,712		

***Significance at the 1% level, **Significance at the 5% level, *Significance at the 10% level.

## Conclusion, Policy Recommendation, and Future Works

### Conclusion

Based on the principal-agent and incentive theories, this study proposes a research model with two management incentives as moderating variables between financialization and the enterprise’s innovation. First, we analyze the direct relationship between financialization and the enterprise’s innovation. Second, we examine the moderating effect of managers’ equity incentive and compensation incentives on the relationship between entity financialization and the enterprise’s innovation in high-tech/non-high-tech enterprises and state-owned and non-state-owned enterprises. The main findings of this paper are as follows. First, the entity financialization will “crowd out” the enterprise’s innovation. Once an enterprise invests in financial assets, the enterprise’s cash flow is positively related to the degree of innovation. The more the cash flows into financial assets, the less the business has liquidity. Companies will spend less on research and development. Therefore, a high rate of return of financial investment leads to the myopia of managers, which leads managers to be more inclined to invest in financial assets, improve the short-term performance of enterprises, and the innovation investment of enterprises will be crowded out. Second, the moderating effect of manager’s incentives is not significant, i.e., manager’s compensation incentive and manager’s equity incentive cannot effectively lower the inhibitory effect of entity financialization on the enterprise’s innovation. The compensation incentive is popular in Chinese companies, but managers prefer to allocate financial assets to obtain more compensation, the governance effect of managers’ compensation incentive will be lowered. The ratio of equity incentive to total incentive is low. Because of the lack of managers’ equity incentives in enterprises, the governance effect of managers’ equity incentives is not significant. Third, compared with non-high-tech and non-state-owned enterprises, the “crowding-out” effect of entity financialization on the enterprise’s innovation is more significant in high-tech and state-owned enterprises. Moreover, the “crowding-out” effect of the manager’s incentive is not entirely achieved. The manager’s incentive mechanism should be improved.

### Policy Recommendation

Entity financialization is a double-edged sword. It could bring significant enterprise profits in the short term, but it also has a negative effect on the enterprise’s innovation. The enterprise’s innovation is the impetus of an entity enterprise’s development, and it is also the enterprise’s most important competitiveness. Based on the above conclusion, four policies and suggestions are recommended below.

Firstly, the balance between financialization and the enterprise’s innovation is crucial. The sound financial investment can alleviate the financing constraints and provide adequate capital for the enterprise’s R&D. However, the entity financialization has a “crowding-out” effect on the enterprise’s innovation. Thus, the appropriate level of financialization should be determined, which benefits the enterprise’s development and competitiveness in the market.

Secondly, the manager’s incentives mechanism in Chinese listed companies should be improved. Currently, the short-term monetary compensation incentive is the essential kind of managers’ compensation in China. But, it will decrease the manager’s tolerance of innovation risk and harm the enterprise’s long-term profit. The proportion of equity incentives should be increased in the manager’s total remuneration, which may positively affect the enterprise’s innovation.

Thirdly, the supervision of managers should be strengthened, and excessive financial investment should be avoided. The managers have absolute power to make decisions on the enterprise’s investment. Thus, the manager’s daily decision-making should be strictly supervised, the manager’s reckless investment, and the loss of enterprise’s profits could be avoided.

Fourthly, financial supervision should be reinforced, and the government should make new financial policies. The financial crisis severely damaged the world’s financial structure in 2008, derived from the over-financialization in the United States. New approaches should be made to prevent over-financialization and also to avoid such financial crises in the future.

### Research Limitations and Future Works

Because of the constraint of research conditions, our studies have three limitations. In addition, we also put forward three future studies.

Firstly, we only analyze the governance mechanism of the effect of entity financialization on the enterprise’s innovation by standing on the perspective of managers’ incentives, some other internal governance mechanisms, such as governance of board directors and governance of larger shareholders, are not taken into account. In future research, we could further study the effect of more internal governance mechanisms on the relationship between entity financialization and the enterprise’s innovation, which could comprehensively elaborate the moderating impact of internal governance on the relationship between financialization and the enterprise’s innovation.

Secondly, entity financialization is regarded as homogeneity; the levels of financialization are not distinguished in this paper. In practice, the impact of managers’ incentives on the relationship between entity financialization and the enterprise’s innovation may be different because of the different levels of financialization. Thus, entity financialization may be divided into two kinds, i.e., over-financialization and non-over-financialization. In future research, the levels of financialization should be distinguished, and it is meaningful to study the effects of entity over-financialization and non-over-financialization on the enterprise’s innovation, respectively, which could exactly describe the relationship between financialization and the enterprise’s innovation.

Thirdly, the mechanism of managers’ incentives is taken as homogeneous. Because the characteristics of managers are also heterogeneous, i.e., different ages, different educational backgrounds, and different genders, they are not considered in this paper. In the future, the characteristics of managers should be introduced into the model and study the effect of managers’ incentives on the relationship between the financialization on the enterprise’s innovation; more accurate results may be attained.

## Data Availability Statement

The original contributions presented in the study are included in the article/supplementary material, further inquiries can be directed to the corresponding author/s.

## Author Contributions

HZ designed the research framework, wrote, and edited the manuscript. CX analyzed the data. MW updated the literature reviews. AI critically worked on research methodology, improve the analysis of the results, and edited the manuscript. All authors contributed to the article and approved the submitted version.

## Conflict of Interest

The authors declare that the research was conducted in the absence of any commercial or financial relationships that could be construed as a potential conflict of interest.

## Publisher’s Note

All claims expressed in this article are solely those of the authors and do not necessarily represent those of their affiliated organizations, or those of the publisher, the editors and the reviewers. Any product that may be evaluated in this article, or claim that may be made by its manufacturer, is not guaranteed or endorsed by the publisher.

## Footnotes

1 BBC News: https://www.bbc.com/news/business-12427321

2 ST is abbreviation of “special treatment.” A stock number with ST means that this company listed in China stock market is in financial trouble or other abnormal conditions. On 22 April 1998, the Shanghai and Shenzhen Stock Exchanges announced to mark special treatment to the stock transactions of listed companies with abnormal financial trouble or other conditions. Such as their stock prices are limited to 5% increase and 5% decrease per day. Moreover, the listed company with *ST keep suffering losses in last 3 years, an early warning of delisting is given. price of the company with*ST is also limited to 5% increase and 5% decrease per day.
